# Metamaterials and Their Application in the Performance Enhancement of Reconfigurable Antennas: A Review

**DOI:** 10.3390/mi14020349

**Published:** 2023-01-30

**Authors:** Musa Hussain, Wahaj Abbas Awan, Mohammed S. Alzaidi, Niamat Hussain, Esraa Mousa Ali, Francisco Falcone

**Affiliations:** 1 Department of Electrical Engineering, Bahria University Islamabad Campus, Islamabad 44000, Pakistan; 2 Department of Information and Communication Engineering, Chungbuk National University, Cheongju 28644, Republic of Korea; 3 Department of Electrical Engineering, College of Engineering, Taif University, P.O. Box 11099, Taif 21944, Saudi Arabia; 4 Department of Smart Device Engineering, Sejong University, Seoul 05006, Republic of Korea; 5 Faculty of Aviation Sciences, Amman Arab University, Amman 11953, Jordan; 6 Electrical Engineering and Communications Department, Universidad Pública de Navarra, Campus Arrosadía, E-31006 Pamplona, Spain; 7 Institute of Smart Cities, Universidad Pública de Navarra, Campus Arrosadía, E-31006 Pamplona, Spain; 8 Tecnologico de Monterrey, School of Engineering and Sciences, Monterrey 64849, Mexico

**Keywords:** metamaterials, metasurfaces, performance improvement, 5G

## Abstract

Metamaterials exhibit properties in terms of subwavelength operation or phase manipulation, among others, that can be used in a variety of applications in 5G communication systems. The future and current 5G devices demand high efficiency, high data rate, computational capabilities, cost-effectiveness, compact size, and low power consumption. This variation and advancement are possible when the antenna design is revised to operate over wideband, high gain, and multiband and has characteristics of compact size, reconfiguration, absorption, and simple ease of fabrication. The materials loaded with antennas or, in the same cases, without antennas, offer the aforementioned characteristics to bring advancement in order to facilitate users. A number of works on designing metasurfaces capable of improving bandwidth, gain efficiency, and reducing the size and cost of antennas are available in the literature for this purpose. Not only are these applications possible, but the intelligent metasurfaces are also designed to obtain reconfiguration in terms of frequency and polarization. The number of absorbers loaded with metamaterials is also designed to improve the absorption percentage used for radar applications. Thus, in this paper, the general overview of different types of metamaterials and their role in performance enhancement and application in 5G and 6G communication systems is discussed.

## 1. Introduction

Wireless technologies emerged with disruptive changes in the late 20th and early 21st centuries. The modifications enabled exponential performance improvement, increased power utilization efficiency, and cost suitability. Swift wireless technologies have also framed the requirements for designing high-performance antennas and propagation systems [1,2]. With further advancements in 5G and future 6G devices, wireless communication systems are required to enable service access to multiple users with cost effectiveness and high data rates. For the aforementioned purposes, the requirements and methodologies of antenna design are also revised [3]. To meet this ever-increasing requirement of current 5G and future 6G communication systems, compact antennas with multiple bands, low profile, high data rate, and ultra-wideband that are geometrically simple for mass production are required [4,5,6].

To attain the aforementioned requirements, several approaches have been studied in relation to antenna and antenna systems, such as stub loading, slot etching, CPW feeding, and defected ground structure (DGS) [7,8,9]. These approaches have been explored in multiple applications, including 5G systems, mm-wave technologies, wireless ON-body devices, and textile applications [10,11,12]. Other approaches employ embedded structures such as electromagnetic band gaps (EBGs) or via ports and pins to enhance antenna performance owing to surface reduction or coupling reduction, among others [13,14,15].

**Figure 1:**
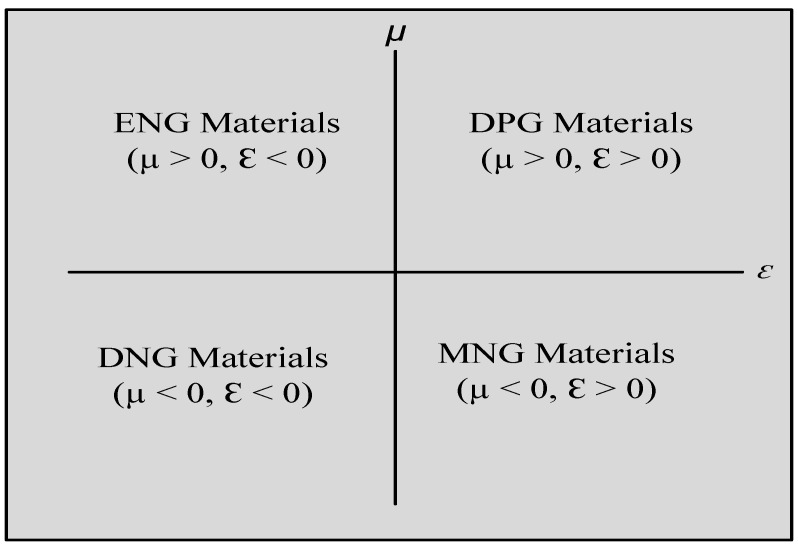
Classification of metamaterial by sign of μ and ε.

With the arrival of the twenty-first century, metamaterials and metastructures are being used on antennas to drastically improve their performance. Metamaterials are artificially designed structures or materials with qualities that vary from those found in nature [16]. The electromagnetic (EM) properties of these materials or mediums are described by electric permittivity (ԑ) or magnetic permeability (μ) [17]. Permittivity and permeability are terms that describe how a material behaves in electric and magnetic fields, respectively. These characteristics might be either positive or negative. When both permittivity and permeability are negative, the material is referred to as a double negative (DNG), which is synthetic and not found in nature. Materials having positive epsilon and mu are referred to as double positive (DPG). These properties may be seen in many plasmas. Furthermore, if the material’s permittivity is positive, it is referred to as epsilon positive (EPG) material. Negative permeability materials, on the other hand, are referred to as μ negative materials (MNGs). Single negative material (SNG) is another name for epsilon or mu negative material [18,19]. Figure 1 depicts these metamaterial categories based on permittivity and permeability.

According to Maxwell’s 1st order differential equation [20]:

(1) ∇×E=−jωB

(2) ∇×H=J+jωD

where *E* refers to an electric field, *H* represents a magnetic field, and ω is the angular frequency. *J* is current density, *B* is magnetic flux density, and *D* is charge density. For simplicity, the above equations can be rewritten as:

(3) ∇×E=−jωμB

(4) ∇×E=−jωμE

**Figure 2:**
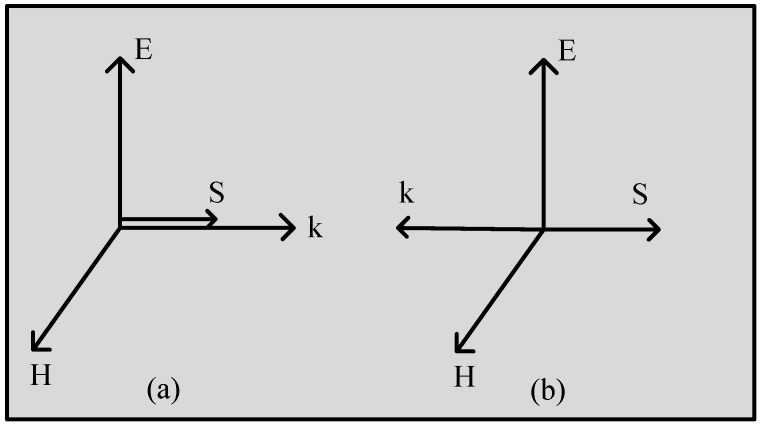
(**a**) Left-handed orthogonal system. (**b**) Right-handed orthogonal system.

For plane waves, the electric and magnetic fields are given by:

(5) $$E=E_{0}\;e^{\left(-jk.r+j\omega t\right)}\;$$

(6) $$H=H_{0}\;e^{\left(-jk.r+j\omega t\right)}\;$$

where *k* is a wave vector and can be expressed with electric and magnetic fields which refer to the orthogonal system, as shown in Equations (7)–(10). The positive value of μ, ε, and *k* makes the right-handed orthogonal system, while negative values make the left-handed orthogonal system as shown in Figure 2.

(7) k×E=ωμH

(8) k×H=ωεE

(9) $$k\times E=-\omega \left|\mu \right|H\;$$

(10) $$k\times H=\omega \left|\varepsilon \right|E$$

In the real part of the Poynting vector, the energy flow is determined.

(11) $$S=\frac{1}{2}E\times H$$

The direction of energy slowing is not affected by changing the sign of μ and ε. Thus, the group velocity is also positive for left- and right-handed orthogonal systems and given as:

(12) $$n=\pm \sqrt{\varepsilon \mu }$$

(13) $$Vp=\frac{c}{n}$$

where “*n*” is the reflective index, *V_p_* is phase velocity, and “*c*” is the speed of light. 

Hence, energy and wave travel in the same direction, which refers to forward wave propagation. In the case of the left-handed system, *n* is negative, implying that VP is also negative, which results in backward wave propagation, which enhances reflected wave power [20]. 

Metamaterials are also known as left-handed materials (LHMs) and are used for several wireless applications [21]. They can be used as an absorber to absorb unwanted radiation and only permit the desired radiation, which can be used for radar and military applications. In addition, they are also used for sensor detections, improving ultrasonic sensors, or many other applications [22,23]. The metamaterials etched on the antenna can improve its performance in terms of bandwidth enhancement [24,25,26,27,28,29,30,31,32,33,34,35,36,37,38,39,40,41,42,43,44,45,46], gain improvement [47,48,49,50,51,52,53,54,55,56,57,58,59,60,61,62,63,64,65,66,67], efficiency improvement [68,69,70,71,72,73,74,75,76], size reduction [77,78,79,80,81,82,83,84,85,86], and isolation improvements [87,88,89,90,91,92,93,94,95,96,97,98,99,100,101,102,103,104,105,106]. In this article, a review of the metamaterial-based antenna is presented, which is used to enhance gain, bandwidth, and efficiency, and reduce the size of the antenna by using frequency-selective surfaces (FSSs), also known as an FSS-based antenna. 

Comparing the present work to other review papers [107,108,109,110], the following are its main contributions:

1. The proposed work provides a summary of the most recent research on the performance improvement of antennas employing metamaterials or structures inspired by metamaterials, presenting state-of-the-art knowledge to researchers.
2. A critical analysis is completed to assist the researcher in fully utilizing the numerous metamaterial-based performance enhancement strategies.
3. In addition, an overview of reflective intelligent surfaces (RISs) and meta-absorbers has been added, which will undoubtedly assist researchers in utilizing these metamaterial properties to further enhance antenna performance.

The rest of the paper contains various sections, which describe the use of different types of metamaterial and their applications in terms of bandwidth enhancement, gain improvement, efficiency improvement, size reduction, reconfigurability of pattern and frequency, and intelligent reflectors and absorbers.

## 2. Performance Enhancement of Antennas by Using Metamaterials

**Figure 3:**
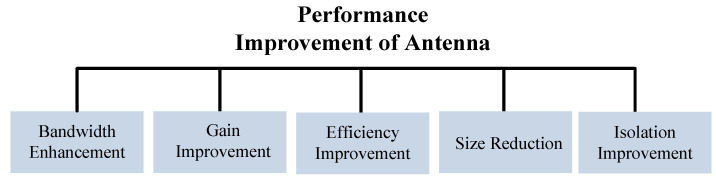
Various performance improvements of antennas by using metamaterials.

The performance of antennas and antenna systems can be enhanced with the aid of metamaterial-based structures, as depicted in Figure 3. The different parameters and approaches followed will be discussed in this section.

### 2.1. Bandwidth Enhancement

Bandwidth of an antenna is a favorable parameter in wireless communication systems. It allows accommodation of exceedingly high data rates for high-performance communication systems [24]. Furthermore, wide bandwidth allows various devices to be concurrently connected to the same band. This enables the connection of various devices, thus improving the overall system efficiency [25]. Wideband antennas are among the hot trend research topics for 5G and future 6G communication systems. Since the systems are gradually improving their requirements of wide bandwidths and high data rates, a shift towards utilization of wideband antennas is inevitable [26]. Owing to this fact, academics and researchers have proposed a number of techniques to enhance bandwidths of antennas [30,31,32,33,34,35,36,37,38,39,40,41,42,43,44,45].

Numerous approaches are used in the literature to increase the bandwidth of antennas; among them, inserting via ports, parasitic patch loading, slot etching, band gap constructions, and stub insertions are some common strategies used by researchers and academics [27]. Even though the use of metamaterial concepts comes with bandwidth enhancement, it also increases the complexity and overall volume of antennas [28]. In this section, the role of metamaterials to enhance the bandwidth of antennas by placing them near/on antennas is reviewed from the literature. 

The principle underlying bandwidth enhancement of an antenna by loading metamaterial is that propagation is zero for zero-order antennas due to miniaturization advantages. The bandwidth and frequency of antennas can be calculated as [29]:

(14) $$\mathrm{Zero}-\mathrm{order}\;\mathrm{resonance}\;\mathrm{frequency}=F_{O}=\frac{1}{\sqrt{LC}}$$

(15) $$\mathrm{Bandwidth}\;\mathrm{of}\;\mathrm{zero}-\mathrm{order}\;\mathrm{resonance}\;\mathrm{frequency}=BW_{O}=G\sqrt{\frac{L}{C}}\;$$

where *G* refers to resistance, *L* inductance, and *C* capacitance. From the above equations, it can be observed that, for bandwidth enhancement, the *G* and *L* should be increased, or *C* decreased. 

The rectangular patch antennas with compact and geometrically simple metamaterial configurations are given in [30,31,32,33,34,35]. The surfaces of a reference antenna are embedded over the front or back side to achieve the requirement of wide band. A new approach in [30] is given to enhance the bandwidth of a microstrip patch antenna. The magneto-electro-dielectric planar waveguided metamaterials (MED-WG-MTMs) are loaded, which consists of a spiral ring resonator (CSR) in the upper metallic plane and a magnetic embedded Hilbert line (EHL) in the ground plane. In the reported design, the operational bandwidth has sharp improvement of 207% at a resonant frequency of 3.5 GHz. 

**Figure 4:**
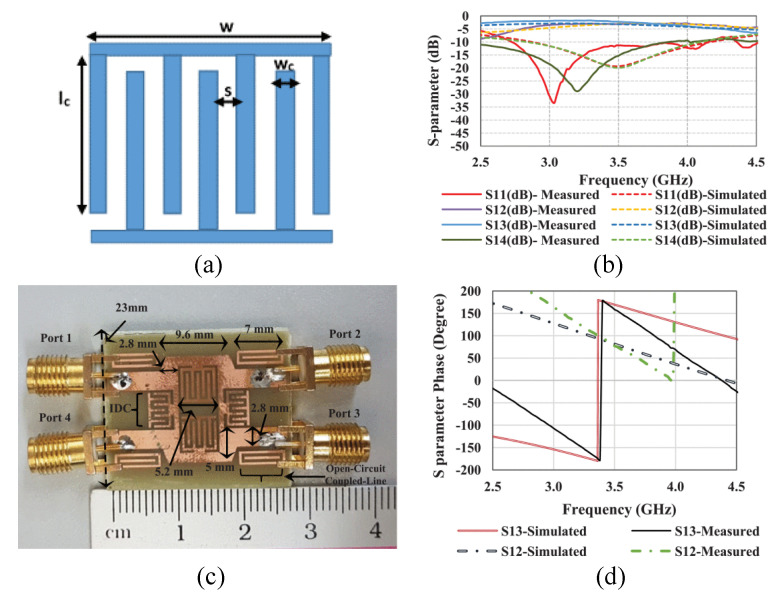
Schematics of the antenna in [27] with metamaterial to improve bandwidth. (**a**) Metamaterial structure, (**b**) S-parameters of the antenna, (**c**) fabricated prototype, (**d**) S-parameter phase. Reprinted from Ref. [27].

In [36,37,38,39,40], a single-layer metamaterial is loaded onto compact size antennas in order to improve the bandwidth. In [36], a bow-tie antenna is reported with integration of a metamaterial lens for the purpose of bandwidth improvement. The antenna shows S11 < −10 dB response from a 300 MHz to 3 GHz frequency. Another work in [37] represents a microstrip patch antenna operating from 3.84–22.7 GHz, as given in Figure 4. The ultra-wideband in the reported work is due to loading of the metamaterial. A planner antenna in [38] for X-band application is reported where an electromagnetic band gap (EBG) structure along with a layer of metallic rings is loaded to improve the bandwidth of 1.6 GHz. In [39,40], microstrip patch antennas loaded with metamaterial are reported to have enhanced bandwidths of 150% and 169%, respectively. 

**Figure 5:**
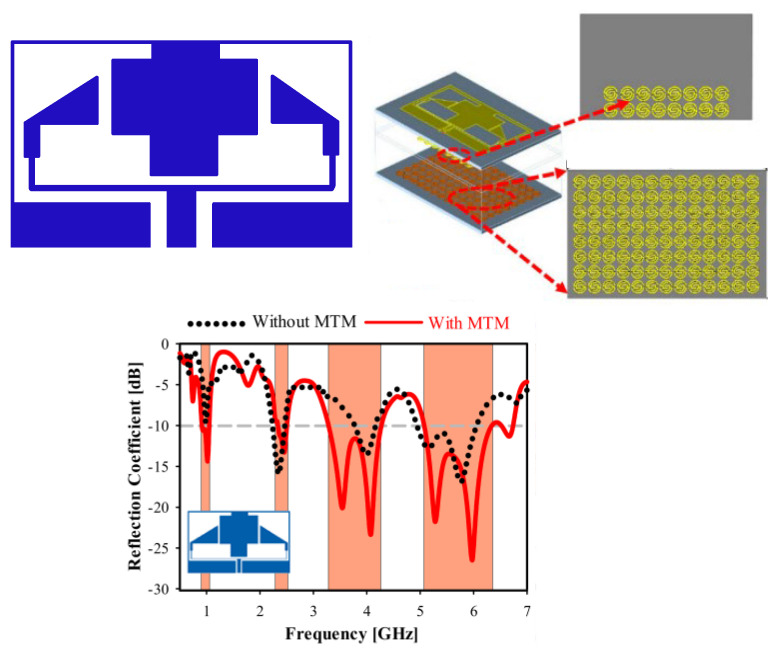
Schematics of the antenna in [43] with metamaterial and return loss. Reprinted from Ref. [43].

In [41,42], a novel geometry of slots is used in the parent antenna integrated with square-like metamaterial at the ground plane in order to enhance the bandwidth of the antenna. For multiband antenna, a metamaterial-based superstrate is utilized to enhance antenna performance in [43], as shown in Figure 5. An overall size of 78.5 mm × 42.5 mm and ultra-wide band of 28 GHz are achieved by placing the surface. The geometry of the antenna consists of two substrate layers. The upper layer has a defected co-planer wave guide-based triangular antenna with a series of slots loaded in the structure. The back side of the antenna and the lower layer both consist of circular metamaterials with concentric slots. The symmetrical C-shaped inner slots of the metamaterial allow control over the surface current density. A stop band is observed at 10 GHz due to opposite flow of the currents. On the contrary, the coherent current at 2.4 GHz and 3.5 GHz allows more depreciation of return loss. Furthermore, various shapes and geometries of metasurfaces are loaded onto compact patch antennas in [44,45,46] to improve the bandwidth of the antennas, in order to achieve a high data rate and throughput to facilitate multiple users, as per demands of 5G and future 6G communication devices.

### 2.2. Gain Improvement

Overlapping between waves leads to interference, in such a way that if two waves with the same frequency, amplitude, and polarization but different phases are propagated in a medium in the z-direction, interference takes place. To enhance gain, this interference must be avoided to obtain a resultant wave with the same phase [25].

Consider two waves with electric fields of:

(16) $$E_{1}=E_{0}\;e^{i\;\left(kx-\omega t-\theta 1\right)}Ž$$

(17) $$E_{2}=E_{0}\;e^{i\;\left(kx-\omega t-\theta 1\right)}Ž$$

as

(18) $$E_{t}=E_{1}+E_{2}=2\;E_{0}[\cos\;(\mathrm{kx}-\omega t-\frac{\theta_{1}+\theta_{2}}{2})\;\cos\;(\frac{\theta_{1}-\theta_{2}}{2})+i\;\sin\;((\mathrm{kx}-\omega t-\frac{\theta_{1}+\theta_{2}}{2})\;\;\cos\;(\frac{\theta_{1}-\theta_{2}}{2})]=2\;E_{0}[\cos\;(\frac{\theta_{1}-\theta_{2}}{2})\;e^{i\;\left(kx-\omega t-\frac{\theta_{1}+\theta_{2}}{2}\right)}Ž\;$$

Here, *E*_0_ shows amplitude while θ phase. By using the Poynting vector:

(19) $${B_{t}}_{\;}=2\;E_{0}[\cos\;(\frac{\theta_{1}-\theta_{2}}{2})e^{i\;\left(kx-\omega t-\frac{\theta_{1}+\theta_{2}}{2}\right)}Ž$$

as

(20) $$S_{t}\;=\;E\;\times \;H=\frac{1}{\mu }\;Bt\times H\;$$

Hence,

(21) $$S_{t}\;=\;4\;E_{0}^{2}\;[\cos^{2}\;(\frac{\theta_{1}-\theta_{2}}{2})\;e^{2i\;\left(kx-\omega t-\frac{\theta 1+\theta 2}{2}\right)}Ž$$

From the above equation, it is clear that energy flow is maximum with phase difference, which refers to the enhancement of gain.

The extensive literature review shows that researchers have made high-gain antennas with the aid of metamaterial structures [47,48,49]. The metasurface placed at the rear end of the antennas acts as a reflector surface. The electromagnetic waves reflected from the metasurface constructively interfere with the propagation waves of the antenna and ultimately result in a high gain response. In addition, metasurfaces also act as lumped inductive and capacitive surfaces which show great response in terms of antenna bandwidth as well as gain [50,51,52,53].

**Figure 6:**
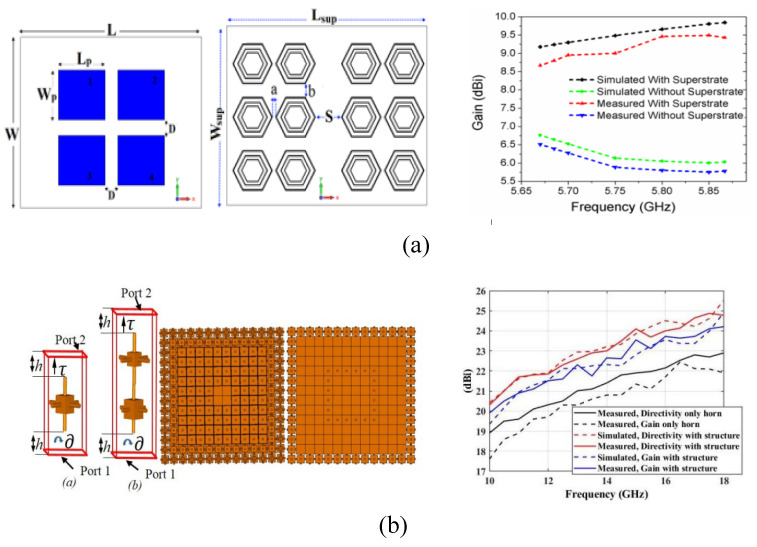
(**a**) Schematics of the MIMO antenna reported in [51] along with gain plot to represent variation in gain with and without metasurfaces. (**b**) Geometry of antenna in [56] with metamaterial and gain improvement plot to verify gain enhancement by single metasurface. Reprinted from Refs. [51,56].

Some metamaterial structures are on the same plane as that of the antenna, etched directly on the same substrate as that of the radiating element [54,55,56]. In this design methodology, the backscattering of the propagation element is minimized and a relatively directional radiation pattern is observed. In [56], the current distribution is varied using two geometrically different metamaterials and two metamaterials with same geometry but different orientations. This completed the required task by making the radiation pattern more directional, thus achieving a higher numerical value of gain as depicted in Figure 6b. 

Conversion of diverging waves into a plane wave and confining of the wave in the antenna aperture also improves the gain of the antenna. The process of confining the waves in the effective area of the antenna can be utilized using a metamaterial lens, as reported in [57,58,59,60,61]. In [57], a two-layer metamaterial lens is placed at a suitable distance from the radiating end of the antenna. The distance between the lens and the antenna is of major importance and set using extensive parametric analysis. The lens confines the diverging waves on the surface of the antenna, thus improving the overall gain of the system. The merit of the presented work is evident from the fact that the gain of the antenna improves from 4.58 dBi to 7.89 dBi (improvement of 70 percent of the value of gain). The geometry of the metamaterial and the relative distance between the radiating body and the metamaterial can bring about the change in the gain of the system.

Terahertz frequency has also gained an extensive design process and methodology. Metamaterials with novel integration of materials such as graphene, SiO_2_, and InSb are utilized for gain enhancement in antennas used for THz applications [62,63]. In [62], a graphene-loaded layer is integrated with a multilayer antenna operating in a THz frequency application, which results in 100% improvement of gain. On another hand, in [63], another THz antenna’s gain is improved from 5.3 dBi to 7.8 dBi by coating near-zero-epsilon metamaterials, that is, SiO_2_ and InSb.

### 2.3. Efficiency Enhancement

Antennas with high radiation efficiency allow more power to be transmitted from the source to the receiver. This allows more power efficiency and more utility of the energy. This parameter is of major consideration for ultra-low-power applications where the energy is a vital consideration. Metamaterials have been utilized for improving the radiation efficiency of antennas as per the reported literature [64,65,66,67,68,69,70,71,72,73,74,75,76]. 

In [64], an implementation of two microstrip lines of zero-order resonator (ZOR) antennas with different heights over the ground plane is demonstrated. The improved ZOR antenna exhibits a far more efficient output of 75% compared to the 10% value of the reference antenna. Monopole antennas loaded with metamaterial for radiation efficiency are reported in [65,66,67]. In [65], a compact antenna with dimensions of 35 mm × 32 mm is presented. Compared with a conventional single band microstrip patch radiator, the radiator size of this antenna is only 8.5% at 2.5 GHz, 17% at 3.5 GHz, and 37% at 5.5 GHz. In addition, the radiation efficiency of the antenna is also pronounced with integration of the metamaterial. Similar work is reported in [66], in which the researchers propose a novel graphene-based plasmonic patch antenna over a metamaterial substrate. The antenna is able to operate in the terahertz spectrum and comprehensive investigation of its radiation efficiency is also presented. The substrate has an anisotropic nature which causes divergence in the fields. Even though this impeding factor is in place, the functionality of the presented antenna has merit since the radiation efficiency is calculated at 16.6%. This numerical value is about four times larger than that of a usual antenna without the metamaterial. 

Ultra-wide band technology has its merits as it can be utilized in low-power application over short distances. Antennas with good radiation efficiency operating in the UWB spectrum have been a topic of research. In [67], a metamaterial structure based on a frequency-selective surface (FSS) cell is proposed to improve the radiation characteristics of the antenna. The antenna has dimensions of 45 mm × 45 mm, and is integrated near an ultra-wide band (UWB) antenna to enhance its performance. The presented work shows polarization mode independency (transverse electric (TE) and transverse magnetic (TM)). With extensive simulations, it is observed that the radiation efficiency of the antenna is enhanced with the FSS filter at a close distance from the radiator. With improvement in the efficiency, the antenna gain is also improved to 3.22 dBi. These performances make the antenna a potential choice for high radiation efficiency applications. A substrate-integrated metamaterial-based leaky wave antenna is proposed to advance radiation bandwidth in [68]. The proposed radiator is designed on a composite right/left-handed substrate-integrated waveguide consisting of two leaky wave radiator elements which are integrated with two-unit cells. The antenna is able to offer beam scanning with frequency from 8.25 GHz to 13.0 GHz. 

**Figure 7:**
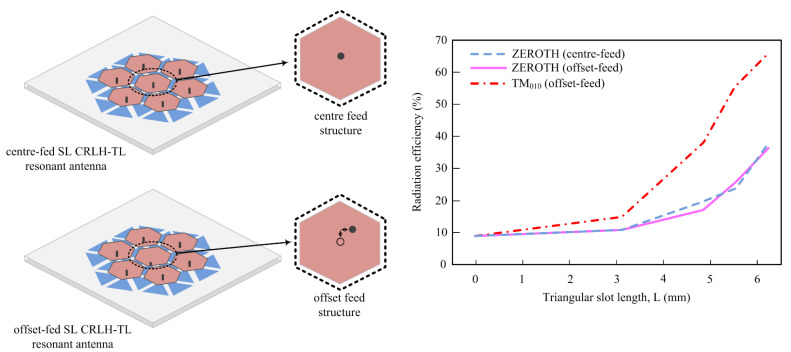
Efficiency enhancement mechanism of metamaterial-loaded antenna (adapted from [69]).

The efficiency improvement metamaterial antenna proposed in [69] consists of center-fed and offset-fed antenna for right- and left-handed transmission linea. In this work, the radiation efficiency is enhanced up to 27.6% in zero-order resonant mode and 56.8% in TM010 mode because of slots etched on the ground plane, as depicted in Figure 7. In [70,71,72], microstrip patch antennas are integrated with metamaterials which enhance the performance of the antennas in terms of their radiation efficiency. The presented designs have split ring resonators affiliated with the radiator or geometrically attached to the metasurface to improve the radiation characteristics. 

### 2.4. Size Reduction

Compact electronic devices are truly a preamble for ubiquitous computing. Communication devices taking up less space and integrated intelligently with computing systems can be incorporated in numerous applications such as navigation and body area networks. Compact antennas have inherent capabilities which allow them to be used at the high end of the frequency spectrum. Thus, high data rates and high transfer of information can be achieved through these antennas. Furthermore, some types of antennas have better radiation patterns when they are electrically small, such as loop antennas. When the radius of the loop increases, the surface current distribution is disturbed, thereby changing the radiation pattern. With the trend of device miniaturization, researchers have proposed a number of antennas with size reduction characteristics [73,74,75,76,77,78,79,80,81,82].

Metamaterials integrated with the radiating structure may offer size reduction. In [73], the antenna contains a unique geometry with a zero-order loop containing a mu negative transmission line achieving low frequency response, whereas a planer circular patch is present in the center of the radiating structure to achieve upper frequency response. 

A microstrip patch antenna is presented in [74] operating in the WIMAX band spectrum. The ground plane of the antenna has a circular slot ring which allows design miniaturization. The LC resonators integrated within the design also allow achievement of a compact size. Such a method is used in [75], in which a z-oriented electric dipole–ENG shell system acts as an LC element. It is important to note that the structure is not integrated on the antenna but surrounds the antenna region. 

In [76,77], metamaterials are integrated into a microstrip patch antenna to reduce the overall dimensions of the antenna. The metamaterial structure is etched onto the radiating patch at the ground plane, providing a capacitive structure which improves the frequency response of the system. Similar work is reported in [78], where a split ring resonator is etched onto the ground plane of the antenna. This behaves as an LC resonator with distributed inductance and capacitance. This electrically small LC resonator also has high-quality factors, thus reducing the power loss in the system.

**Figure 8:**
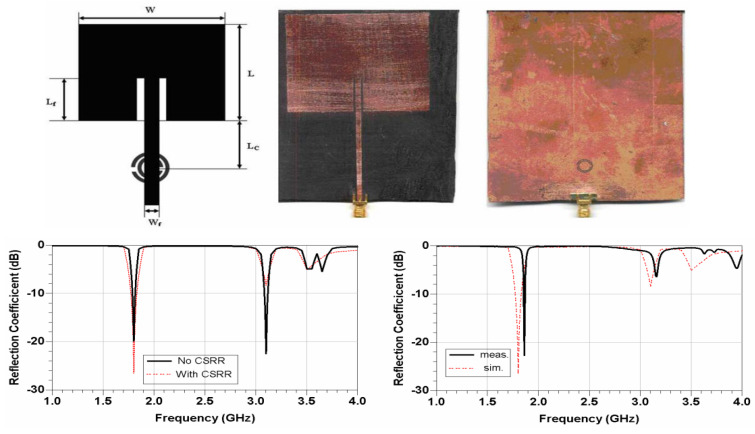
Return loss comparison of CSRR-loaded antenna proposed in [79], representing the size reduction of the antenna. Reprinted from Ref. [79].

Microstrip patch antennas utilize geometric variation in the parent patch and integrate the split ring resonators in the ground plane which generally improve the dimensions of the antenna [79], as given in Figure 8. It can be observed that no net effect on the simulated and measured S-parameter is observed by loading CSRR, whereas size is reduced in order to obtain a compact antenna. In [80], the comparison of antenna characteristics in terms of physical size, reflection co-efficient, bandwidth, gain, and radiation patten is performed, which shows that by loading metamaterial onto antenna, the size of the antenna is reduced by 28% and up to 70% in the case of the half- and full-ground plane, respectively. A metamaterial-loaded antenna for size reduction applications is given in [81], which shows that after loading the metamaterial, the antenna’s size was reduced by 82.77% with 38.23% improvement in return loss. Moreover, in [82] an antenna designed on a high-permittivity dielectric superstrate is given, where a periodic structure of patches acting as an AMC is used for size reduction of the antenna.

### 2.5. Isolation Improvement

In current and future advancements of technology, high data rates and high-quality services for multiantenna system are required. Due to close placement of antennas, the issue of mutual coupling, also known as isolation, arises [83]. To order to improve the isolation of MIMO antenna systems, the power radiation is reduced, which can be carried out to improve the distance between two elements. This method results in increased size, and cannot be used in future devices as compactness is one of the key parameters for future communicating devices [84,85]. Recently, the metamaterial structure has been loaded onto antennas in order to improve isolation and maintain a compact size and low complexity [86].

**Figure 9:**
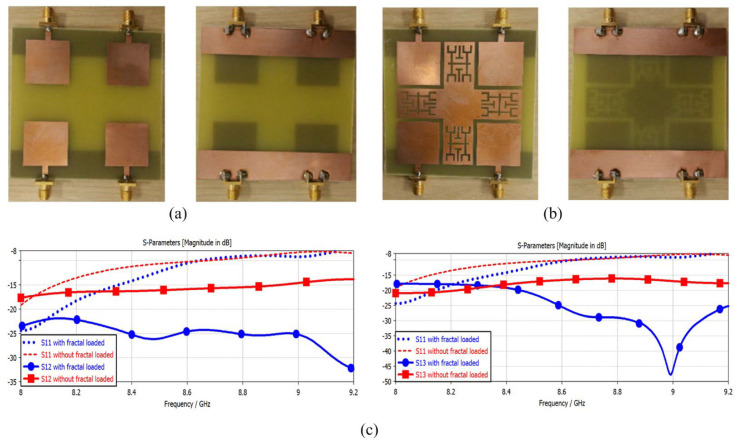
Fabricated prototype of MIMO antenna (**a**) without decoupling structure, (**b**) with decoupling structure. (**c**) S-parameters of MIMO antenna with and without structure. Reprinted from Ref. [90].

The work reported in [87] shows that a 3D novel metamaterial structure was used to improve the isolation from 14 dB to 18 dB of MIMO antennas. The isolation of closely placed MIMO antennas in [88] is improved by 23 dB as compared to the original case. A metamaterial with positive and negative permeability is loaded onto the antenna to reduce mutual coupling to MIMO elements. The metamaterial can be loaded between two or more MIMO elements to improve isolation [89,90], this structure is also known as an electromagnetic band gap (EBG) structure. In this method, one of the radiating elements in the array is excited which causes a surface wave to spread out and also induce current, which results in a reduction in mutual coupling between antennas [91]. The antenna reported in [90] with and without a metamaterial structure and its effect on transmission as well as reflection coefficient are given in Figure 9.

**Figure 10:**
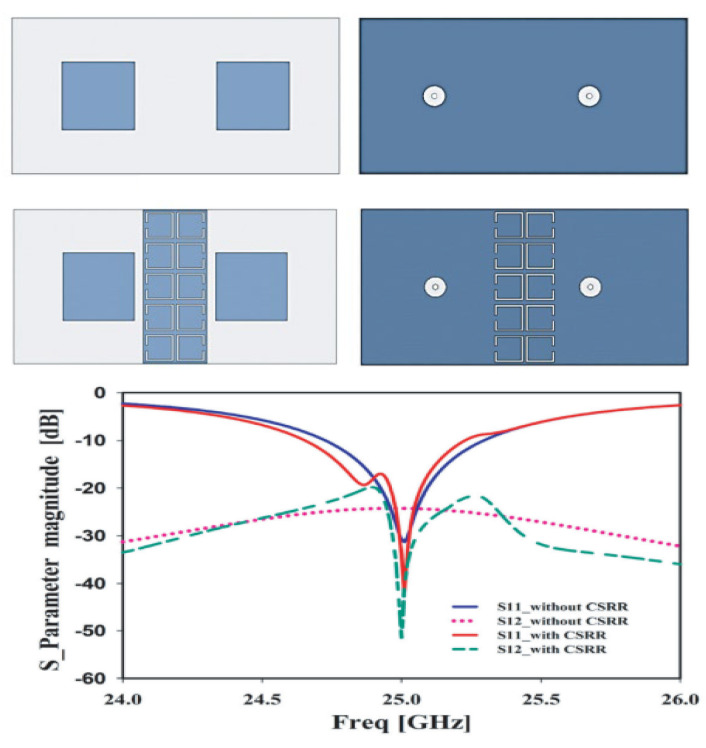
Top and bottom view of antenna with and without decoupling element and the effect of insertion of CSRR to enhance isolation. Reprinted from Ref. [95].

Metamaterial absorbers are also widely used in order to reduce mutual coupling of MIMO antenna systems. A 4 × 2 array of metamaterial absorbers is placed between two PIFA elements in order to improve isolation to 80 dB at the operating band of the MIMO element [92,93]. Complementary split ring resonators (CSRRs) are also used as a metamaterial to improve the isolation of MIMO antennas. The fundamental reason to build a CSRR (which is basically a decoupling structure) is that it exhibits strong characteristics of band rejection, and can be implemented as a cascaded stopband filter and used for isolation enhancement purposes [94,95,96,97]. In [95], the isolation is enhanced to 25dB as compared to a simple array design, as shown in Figure 10.

In [98], an interdigital split ring resonator (SRR) with negative permeability (which is known as MNG metamaterial) is introduced between radiating elements to reduce mutual coupling and enhance isolation of MIMO antenna systems from 17 dB to >25 dB. The metamaterial structure placed between radiating elements in [99], where there is no short circuit ground plane using via holes, achieves around 60 dB isolation in a four-element array design.

In radar applications, metamaterial absorbers (MAs) are widely used to reduce radar cross section (RCS) and improve isolation. In [100], an MA is used to improve the isolation to 34 dB and small value of RCS of 11 dB is achieved. Thus, the MA and SRR are widely used between resonating elements to improve the isolation and reduce the mutual coupling [101]. 

## 3. Reconfigurability with Metamaterials

**Figure 11:**
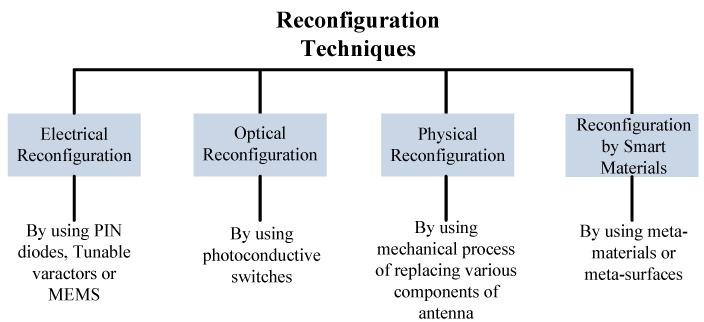
Various techniques adopted to obtain reconfiguration.

Modern wireless communication systems require antennas to operate in multiple modes used for different wireless services. These multiple mode operating antennas need fast and efficient systems to provide shifting among various modes, which can be true in reconfigurable antennas [102]. The reconfiguration of antennas relates to the capacity to adjust radiator performance in terms of operating frequency, radiation pattern, or polarization [103]. A number of techniques are adopted to obtain the requirements discussed above by researchers and academia. Reconfigurations are achieved by using p-i-n diodes, MEMS, tunable varactors, optical devices, or mechanical techniques as given in Figure 11 [104]. In this section, the reconfiguration of antennas by using metamaterials will be discussed in term of frequency, pattern, and beam steering.

### 3.1. Antenna Pattern Reconfigurability

The requirement of pattern reconfiguration in antenna systems for the current 5G and future 6G is increased due to multipath fading. This phenomenon occurs more predominantly in urban areas due to multiple reflections and scattering of signals [105]. The large numbers of users with limited-frequency bandwidth increase interference in cities and crowded areas. The pattern-reconfigurable antenna overcame this problem due to its diverse functions [106]. In addition, the reconfigurable antenna also saves installation space and fabrication cost, as a single antenna can achieve the performance of multiple antennas [111].

**Figure 12:**
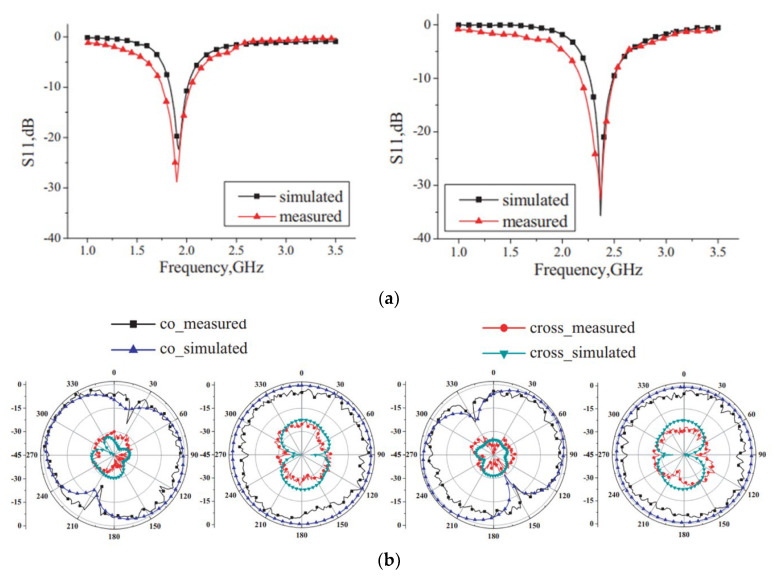
(**a**) Comparison among simulated and measured S-parameters; (**b**) radiation pattern for resonating frequencies. Reprinted from Ref. [105].

A metamaterial-loaded dipole antenna operating over a 28 GHz application is given in [112], which provides a 28° main beam deflection after loading CSR metamaterial. A tri-band antenna offering 1.5 GHz, 2.4 GHz, and 3.3 GHz is reported in [113]. The reported antenna is loaded with a CSRR metamaterial transmission line which provides 45° pattern shifting in three different modes. A pattern reconfiguration of a loop antenna is studied in [114], where a physical metallic strip is loaded to achieve reconfiguration. The reported work is given in Figure 12, where it can be observed that the antenna consists of a loop-based periodically loaded capacitive arc strip and a pair of independent ports with hybrid feeding networks. It can be seen from the figure, in the case of port 1, that a horizontally polarized omni-directional pattern is obtained for odd mode radiation in the xy-plane, while it is figure of eight-shaped in the yz-plane. In the case of port 2, the good omni-directional pattern in the yz-plane is given. 

A Huygens dipole antenna is reported in [115] with a low profile, compact size, and high gain of 5.3 dBi operating at 1.5 GHz. The reported work involved a reconfigurable element and two pairs of electric and magnetic near-field parasitic (NFRP) elements. The reconfigurable element contains a pair of p-i-n diodes in order to perform pattern reconfiguration. The antenna offers two independent uni-directional end-fire radiating states by switching the p-i-n diodes on or off. The peak point of radiation is in antipodal directions and bi-directional end-fire radiating state. The metamaterial-loaded antenna can be used for a side-lobe cancelation mechanism, which is widely used in smart vehicle applications [116]. Magnetic photonic crystals (PCs) are also used to control the radiation pattern of antennas, known as the optical reconfiguration method [117]. In [118], an antenna operating at ultra-high frequency (UHF) is reported for biomedical applications. The metamaterial layer contains p-i-n diodes to provide pattern reconfiguration from −30° to +30°. Another antenna for on-body applications is reported in [119]. The antenna has simple geometry and covers the 2.4 GHz band for ISM applications. The pattern reconfiguration is achieved by loading metamaterial and the results are verified by conformal analysis as well. 

A reconfigurable antenna has the additional advantage of freedom of degree and multifunctionalities. Reconfigurable radiation pattern antennas are free to radiate in any desired direction, which is further made easy by metasurfaces and metamaterials [120,121]. The knowledge to perform pattern reconfiguration in metamaterial is also obtained by using various programable algorithms by using FPGA [122]. The broadband and low-profile antenna loaded with mushroom-type metamaterial in [123] provides five states of reconfiguration. 

The compact and geometrically simple antenna loaded with metamaterial reported in [124] offers pattern reconfiguration. The metamaterial contains two p-i-n diodes to provide four different radiation patterns. Besides rigid materials, the pattern-reconfigurable antennas are also based on flexible substrate materials with compact size and simple geometry [125]. Metamaterial loaded on the ground plane of the antenna and connected with a radiator by a via port is commonly used to provide pattern reconfiguration [126]. An interesting work is reported in [127], where the antenna is connected with a metamaterial structure placed on the ground plane to provide pattern reconfiguration. The antenna has a compact size and simple geometry offering narrow- as well as broadband.

### 3.2. Antenna Frequency Reconfigrability

As for pattern reconfiguration, the antennas became operational for frequency reconfiguration by adopting aforementioned techniques. Frequency reconfiguration is also obtained by the traditional method of inserting p-i-n diodes, but in current 5G and future 6G communication systems, the metasurfaces should be loaded onto antenna in order to achieve the frequency reconfiguration [128]. One of the key advantages of this approach is protecting the antenna or communicating devices from heat. The diode inserted in the antenna to perform reconfiguration heats up, which damages the device. Moreover, the size of the antenna is reduced, cost is reduced, and structural complexity is also reduced by loading metamaterials instead of using other techniques [129,130,131].

The propagation of electromagnetic waves is suppressed at the band gap by using a high-impedance surface (HIS) metamaterial known as an electronic band gap (EBG). The bandwidth of the EBG unit cell is referred to as a band gap, which is implemented to suppress the frequency at which the antenna works while, on other hand, it allows other frequencies [132]. In [133], a bridge-shaped resonator (BSR) is placed on the front side while a strip line is placed on the back side of a unit cell metamaterial. Four switches are added in the gap structure to obtain reconfigurability from 28 GHz to 36 GHz. A metamaterial-inspired frequency-switchable antenna having simple geometry and compact size is reported for 1.6–2.23 GHz applications in [134]. The concept of NRI metamaterial is adopted to achieve dual bands and varactor diodes are inserted to obtain reconfiguration. A low-profile antenna operating on dual bands of 3.2–4 GHz and 4.4–5.8 GHz is reported in [135]. The reported antenna contains two feeding structures placed vertically with branches used as a filter as well as an artificial magnetic conductor (AMC). The p-i-n diodes are introduced to obtain the reconfiguration.

A metamaterial-loaded antenna with modified fractal ground plane is reported in [136], which provides reconfigurability as well as performance enhancement. Another metamaterial-inspired frequency-reconfigurable antenna is reported in [137]. The antenna has a circular complimentary split ring resonator (CCSRR) and hexagonal complimentary split ring resonator (HCSRR). Two switches are loaded onto antenna, which are used to provide reconfiguration between dual bands of 3.95 GHz and 5.72 GHz. Two-layered metamaterials are also used to improve the performance of antennas in future 5G and 6G communication systems. In [138], the antenna carries a slot-coupled patch and two-layered metasurfaces. The upper layer is partially integrated with the absorbing surface while the bottom layer contains a tunable phase cell. This antenna operates in the 8–14 GHz band spectrum with a high peak gain of 7dBi. Another dual-layer metamaterial-inspired antenna is reported in [139]. The reported work describes frequency reconfiguration as well as polarization reconfiguration by using double-layer metasurfaces.

**Figure 13:**
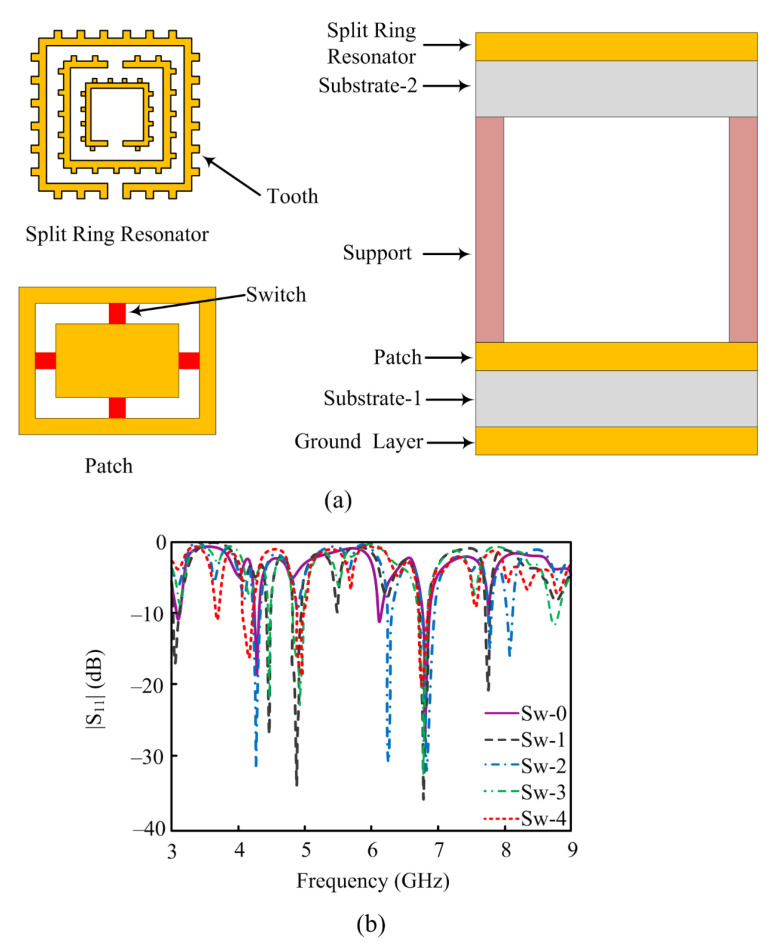
The SRR loaded reconfigurable antenna (**a**) geometrical configuration (**b**) |S_11_| results (adapted from [143]).

In the literature, a novel approach is adopted by considering liquid in metamaterial [136,137,138,139,140] in order to achieve various levels of performance enhancement or reconfiguration or both. The antennas are made with traditional approaches for tuning frequency by using p-i-n diodes, spring resonators, via ports, or EBGs as given in [140,141,142]. In [143], copper- and seawater-based SRRs are used to verify the reconfigurable results of the proposed work. In order to obtain better reconfiguration, a tooth is added, which also improves the gain of the antenna. The reported work is operational over 3–9 GHz frequency bands by using five different tunable modes. Figure 13 depicts the geometry of reported the antenna as well as the placement of switches used for reconfiguration. Five different switching modes of turning switches on or off are applied to the antenna.

### 3.3. Intelligent Reflecting Surfaces

Reconfigurable intelligent surfaces (RISs) are flat surfaces which are capable of manipulating the phase and wavefront of incoming radio waves that need to be controlled. This improves the reception of the electromagnetic signals, generally through maximization of energy efficiency and optimal transmit beam forming by consideration of the propagation environment [144,145]. RISs are electromagnetically disconnected and may consist of subcomponents, such as capacitors and diodes, influencing the signals in a way that is not possible naturally [146]. 

Energy efficiency is one of the key parameters in propagation of electromagnetic waves. RIS technology is very useful in this case because it allows the amplification of incoming signals using a pattern of constructive interference of a number of signals using appropriate phase shifts from each of the reflecting elements. This methodology mitigates the need for an electronic power amplifier for amplification purposes, thereby reducing the power consumption [147,148]. A number of design methodologies are utilized based on intelligent surfaces. Some of them utilize electrical elements, such as diodes integrated with a metamaterial. These surfaces are capable of varying the phase and scattering properties of the radiated wave. Hypersurfaces are the most recent technology which are controlled by an embedded software. These surfaces allow beam steering, polarization variation, and phase shifting for the optimization of beam transmission [149,150,151,152]. 

**Figure 14:**
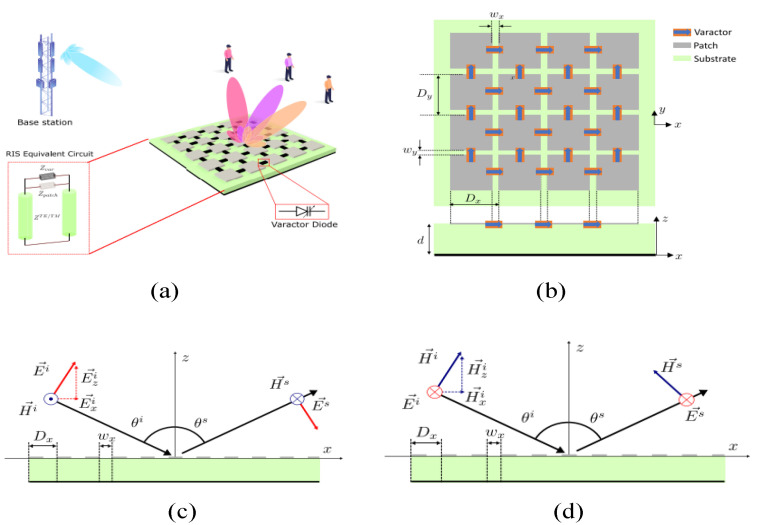
(**a**) Layout of RIS surfaces. (**b**) Geometry of reported intelligent surfaces loaded with diodes. (**c**) Parallel polarization. (**d**) Perpendicular polarization. Reprinted from Ref. [153].

The EM model for reconfigurable intelligent surfaces is given in [153] and depicted in Figure 14a,b. According to the reported work, a number of ways are adopted to obtain full reconfigurability but the most simple and convent method is using varactor diodes in each unit cell of the intelligent surface. This figure also exhibits the perpendicular and parallel incidence polarization. In the case of parallel polarization, the electric field and incidence are in the same plane while the magnetic field is orthogonal. In the case of perpendicular polarization, the magnetic field and incidence are in the same plane while the electric field is orthogonal, as depicted in Figure 14c,d.

The aforementioned discussion made it clear that IRS is a guided and well-trained metasurface used for various applications in future 5G and 6G communication. Besides many other applications, one key application in the improvement in rank of MIMO communication, also called channel capacity enhancement, is reported in [154]. In traditional MIMO systems, the channel capacity is obtained by spatial multiplexing, which is larger in line-of-sight (LOS) cases only. By implementing IRS, the spatial multiplexing is enhanced in LOS as well as non-LOS systems. 

Intelligent reflecting surfaces have a number of applications, but there are also some research gaps noticed by researchers and academia in the literature. Some of the research challenges are: channel tracking in RIS-empowered networks, designing RIS-empowered EM wave propagation, modeling of passive and active RIS architectures, algorithms for RIS-enabled EM wave control, channel state acquisition, hybrid transceivers for mm-wave IRS-assisted systems, near-field region and spherical wavefront mode, user balancing and scheduling, and many more [155,156].

### 3.4. Metamaterial Absorbers

The trend of working on metamaterials and metasurfaces gives new hope for designing absorbers due to their unique properties in term of values of permittivity and permeability, which are not found in materials existing in nature. In 2008, the first ever experiment was performed on designing a novel metamaterial perfect absorber (MMPA). Later on, single-band, multiband, and broadband metamaterial absorbers were designed for various wireless applications. The most important factor when using a metamaterial absorber instead of a classical absorber is thickness, as it is 25 times smaller than that of other ones [157,158,159,160]. Recently, a metamaterial absorber was designed for future 6G application, operating on the terahertz (THz) frequency spectrum with numerous properties of multiband, wide band, and polarization diversity [161,162]. Afterward, a sensor-based metamaterial absorber was also introduced having advantages of low cost, high sensitivity, easy fabrication, and good quality factor. These absorbers can be used to measure pressure, temperature, and density and to determine EM properties of materials [163].

The artificially engineered metamaterials consist of a sequenced array of conducting metal and dielectrics having accommodating permittivity and permeability for incident waves [164,165]. In [166], an ultra-wide band metamaterial-based absorber is reported for 1.4–6 GHz application, covering GPS, ISM, WLAN, WiMAX, 5G sub-6GHz, and C-band spectrums. The absorber was designed by using resistive sheets and has absorption over 96% in the operational band. 

**Figure 15:**
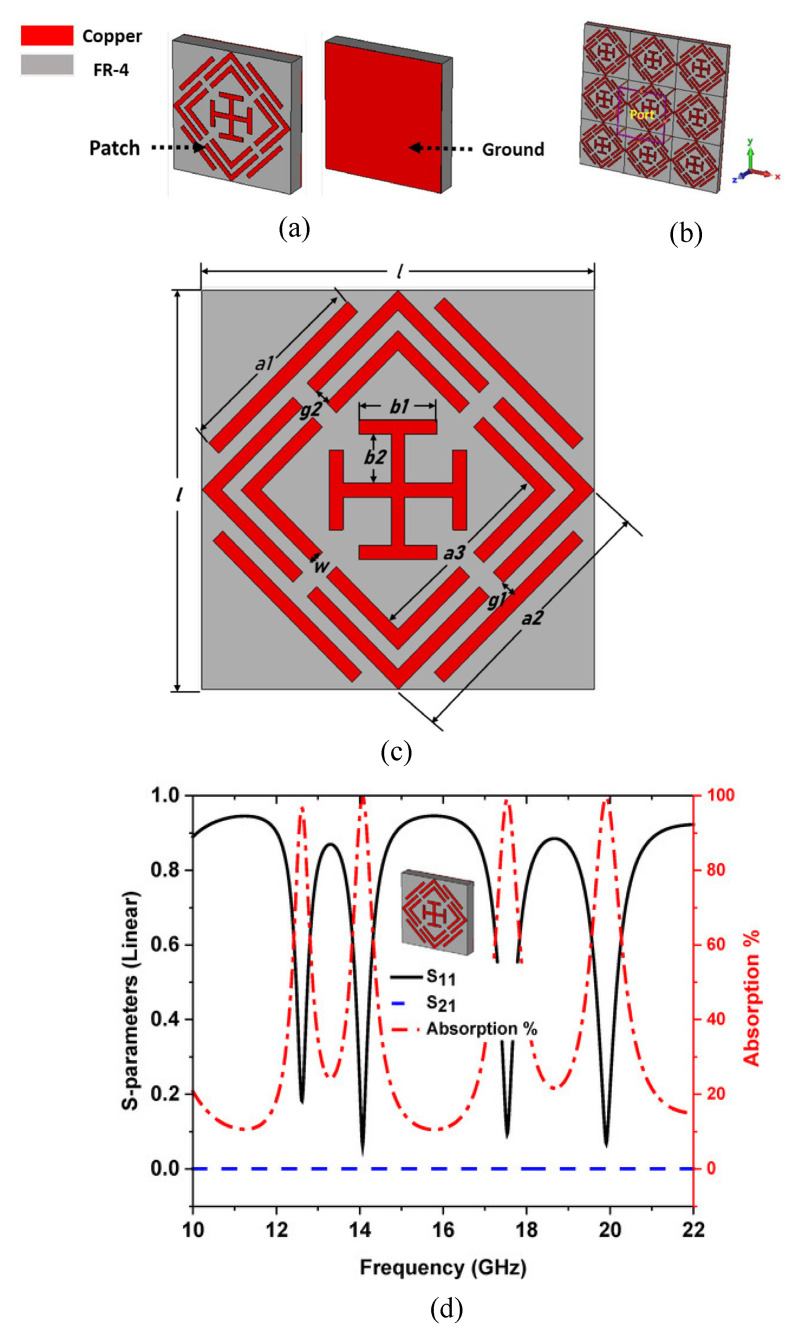
(**a**) Single cell of the absorber (**b**) 3 × 3 array of the absorber (**c**) geometry of reported metamaterial absorber. (**d**) S-parameter plot along with absorption percentage of reported SSRR-loaded metamaterial absorber. Reprinted from Ref. [167].

In [167], a quad-band metamaterial absorber is presented for Ku and K band sensing applications. The presented absorber contains an SSRR and operates over 12.62 GHz, 14.12 GHz, 17.53 GHz, and 19.91 GHz with 97%, 99.51%, 99%, and 99.5% absorption, respectively. The metallic portion contains a snake-shaped periodically arranged pattern as given in Figure 15a. The absorber is designed over an FR-4 sheet having a Teflon layer with total thickness of 2.44 mm. The overall size of the reported sheet is 50 cm × 50 cm. The results in term of absorption percentage, impedance versus frequency, and parametric analysis of various thicknesses of the dielectric layer and its effect on absorption are reported in Figure 15b. 

Besides the aforementioned advantages and properties of metamaterial absorbers, in the literature a number of frequency-tunable metamaterial absorbers are also reported. The reconfigurability of the absorber is obtained by placing varactor diodes in metallic shapes [168]. In [169], a frequency- and bandwidth-switchable metamaterial absorber is designed for X-band applications. Each unit cell in the reported absorber has a varactor diode in the center of the microstrip line resonator. The unit cell has dimensions of 16 mm × 10 mm with thickness of 0.8 mm. It is designed on the top side of an FR4 sheet having 20 × 14 unit cells and it covers a wideband of 8–12 GHz. The frequency and absorption comparison is given in the figure, where the bias voltage and capacitance of the varactor are varied to show the reconfigurability of the reported metamaterial-based absorber for X-band applications.

**Figure 16:**
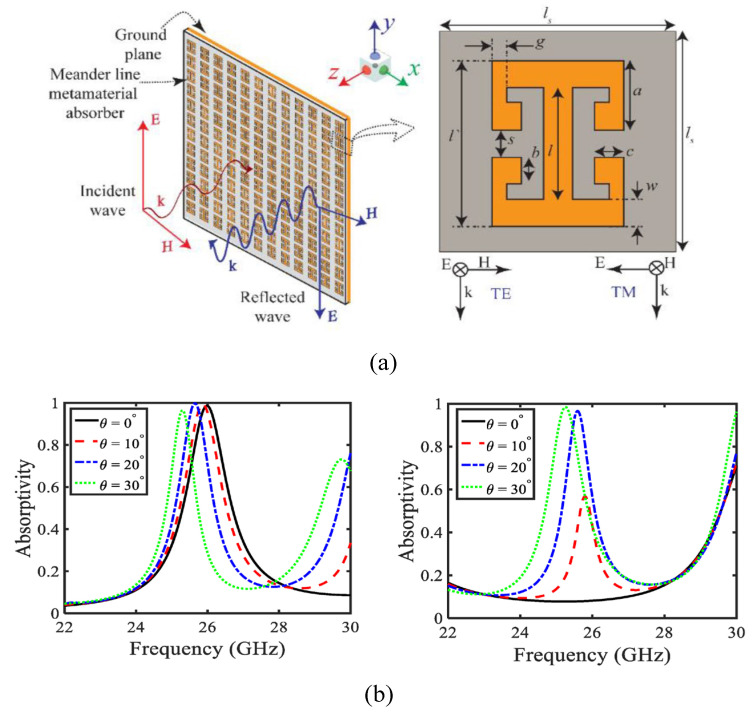
Dual-band absorber with geometry snapshot and absorption percentage plot (**a**) geometrical configuration (**b**) absorption results. Reprinted from Ref [170].

In [170], a novel meander line metamaterial absorber is reported for millimeter-wave applications. The reported absorber operates at 24 GHz and 28 GHz and offers an absorption percentage >99.5% at operational bands. The geometry and absorption percentage at various angles are given in Figure 16.

**Figure 17:**
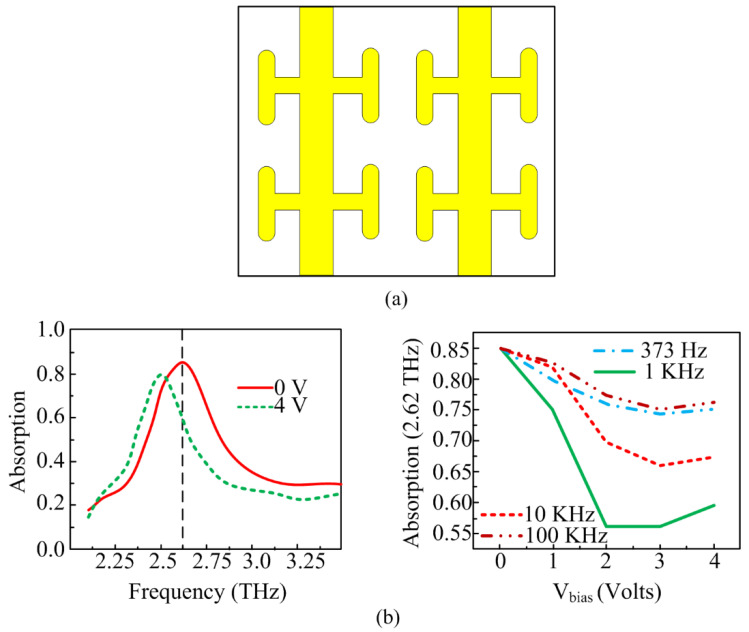
(**a**) Unit cell geometry of reported liquid crystal-based metamaterial absorber. (**b**) Absorption percentage of reported absorber in which liquid crystals are either aligned or not (adapted from [172]).

A tunable graphene-based metamaterial absorber for terahertz applications is reported in [171]. The unit cell has a cross-shaped resonator and double-layer graphene wires. The reported absorber has the advantage of electrical control of absorption and more flexibility in polarization states of THz. One other work is reported in [172] and depicted in Figure 17. The reported metamaterial absorber operates over the 2.25–3.25 THz spectrum. The absorption of the unit cell is modified by 30% at 2.62 THz and bandwidth improvement of 4% is seen due to the location of liquid crystals. It is a novel approach to obtain tunability by insertion of liquid crystals. The metamaterial absorber has absorption of >80% at operational bandwidth. The schematic of the unit cell, liquid crystal, and its placement along with results in terms of absorption is given in Figure 17. 

## 4. Conclusions

In this review paper, a comprehensive review of metamaterial and its application in 5G and 6G communication systems is given. The application of metamaterials includes the enhancement of bandwidth and gain, size reduction, efficiency enhancement, reconfiguration of frequency and pattern, and intelligent reflecting surfaces and absorbers. For bandwidth, gain, and efficiency improvement, the metamaterials are loaded in the front or back of the reference antenna to obtain the desired improved results. In the aforementioned discussion, examples from the literature are given to show the workings and results of metamaterial-loaded antennas. For size reduction, the metamaterial is placed on the same plane as the antenna to obtain a miniaturized antenna with the same results as a larger antenna. Afterward, the reconfiguration application of metamaterial is discussed in terms of frequency reconfigurability and patter reconfigurability, along with various examples from the literature. Then, the current trend of metamaterial is discussed in terms of its applications in intelligent selective surfaces and absorbers. Researchers propose a number of studies, but only a few interesting articles are reported in this paper to serve as examples and evidence.

## Data Availability

Not applicable.
